# Endothelial nitric oxide synthase (eNOS)-NO signaling axis functions to promote the growth of prostate cancer stem-like cells

**DOI:** 10.1186/s13287-022-02864-6

**Published:** 2022-05-07

**Authors:** Weijie Gao, Yuliang Wang, Shan Yu, Zhu Wang, Taiyang Ma, Andrew Man-Lok Chan, Peter Ka-Fung Chiu, Chi-Fai Ng, Dinglan Wu, Franky Leung Chan

**Affiliations:** 1 State Key Laboratory of Oncology in South China, Collaborative Innovation Center for Cancer Medicine, Sun Yat-Sen University Cancer Center, Guangzhou, China; 2 School of Biomedical Sciences, The Chinese University of Hong Kong, Shatin, Hong Kong, China; 3 Department of Surgery, Faculty of Medicine, The Chinese University of Hong Kong, Hong Kong, China; 4 Shenzhen Key Laboratory of Viral Oncology, The Clinical Innovation & Research Center (CIRC), Shenzhen Hospital, Southern Medical University, Shenzhen, Guangdong China

**Keywords:** Prostate cancer, Castration resistance, Prostate cancer stem-like cells, ERRα, ERG, eNOS, NO

## Abstract

**Background:**

Accumulating evidence supports that prostate cancer stem-like cells (PCSCs) play significant roles in therapy resistance and metastasis of prostate cancer. Many studies also show that nitric oxide (NO) synthesized by NO synthases can function to promote tumor progression. However, the exact roles of NOSs and NO signaling in the growth regulation of PCSCs and castration-resistant prostate cancer (CRPC) are still not fully understood.

**Methods:**

The regulatory functions of NOS-NO signaling were evaluated in prostate cancer cells, especially in PCSCs enriched by 3D spheroid culture and CD133/CD44 cell sorting. The molecular mechanisms of NOS-NO signaling in PCSCs growth regulation and tumor metastasis were investigated in PCSCs and mice orthotopic prostate tumor model.

**Results:**

Endothelial NOS (eNOS) exhibited a significant upregulation in high-grade prostate cancer and metastatic CRPC. Xenograft models of CRPC exhibited notable increased eNOS expression and higher intracellular NO levels. PCSCs isolated from various models displayed significant enhanced eNOS-NO signaling. Functional analyses demonstrated that increased eNOS expression could promote in vivo tumorigenicity and metastatic potential of prostate cancer cells. Characterization of eNOS-NO involved downstream pathway which confirmed that enhanced eNOS signaling could promote the growth of PCSCs and antiandrogen-resistant prostate cancer cells via an activated downstream NO-sGC-cGMP-PKG effector signaling pathway. Interestingly, eNOS expression could be co-targeted by nuclear receptor ERRα and transcription factor ERG in prostate cancer cells and PCSCs.

**Conclusions:**

Enhanced eNOS-NO signaling could function to promote the growth of PCSCs and also the development of metastatic CRPC. Besides eNOS-NO as potential targets, targeting its upstream regulators (ERRα and ERG) of eNOS-NO signaling could also be the therapeutic strategy for the management of advanced prostate cancer, particularly the aggressive cancer carrying with the *TMPRSS2:ERG* fusion gene.

**Supplementary Information:**

The online version contains supplementary material available at 10.1186/s13287-022-02864-6.

## Introduction

It is well recognized that many cancers, including prostate cancer, contain heterogeneous populations of transformed cells, with difference in their growth features and clinical behaviors. Accumulating evidence shows that within cancers, there is a small subpopulation of highly tumorigenic cancer cells designated as cancer stem-like cells (CSCs; also called tumor initiating or cancer progenitor cells), thus named largely due to their stemness features, such as expression of stem cells-associated transcription factors, high self-renewal capacity and phenotypic plasticity [1]. Experimental studies indicate that these CSCs, characterized by their high tumor-initiating potential, can participate in tumor relapse, metastasis and therapy resistance [2, 3]. Hence, targeting the CSCs-associated signaling pathways or regulators has become an attractive potential therapeutic strategy for treating advanced therapy-resistant cancers or possibly their eradication [1, 4, 5].

Studies in prostate cancer validate that prostate cancer stem-like cells (PCSCs) can be indeed isolated from various sources, including prostate cancer cell lines and patient-derived tumor xenografts, using different methodologies [6]. Emerging evidence suggests that PCSCs could play crucial roles not only in tumor initiation but also advanced malignant progression to castration-resistance and metastasis, likely related to their insensitivity to androgen deprivation therapy and phenotypic plasticity [7–9]. Current advances reveal that PCSC-conferred castration and chemotherapy resistance involve multiple and cross-interacting signaling pathways, including dysregulated AR signaling and androgen metabolism, dysregulated epigenetic and miRNA control, aberrant activated signaling pathways as regulated by growth factor receptor tyrosine kinase (PI3K/AKT), PTEN, STAT3, WNT/β-catenin, NOTCH, SHH, TGFβ and NF-κB [10–12]. Few studies in different experimental models suggest that targeting certain CSC-associated pathways, including Notch, hedgehog, WNT/β-catenin, STAT3 and TGFβ, can suppress the cancer stemness or attenuate the CRPC progression [13–17]. Recently, we have established an immunotherapeutic platform targeting PCSCs, based on sensitization of dendritic cells–cytokine-induced killer cells by PCSC-derived immunogenic peptides [18]. These encouraging studies suggest that targeting PCSCs or their possible eradication would be an attractive therapeutic strategy for improved therapy of advanced therapy resistance and metastatic prostate cancer.

Nitric oxide (NO) is an important multi-functional gaseous cellular signaling regulator, being implicated to perform roles in different stages of cancer progression, particularly inflammation, angiogenesis and metastasis, and also therapy resistance [19, 20]. Intracellular NO can be synthesized by either one of the three nitric oxide synthase (NOS) isoforms using L-arginine, NADPH and oxygen: including neuronal (nNOS/*NOS1*), inducible (iNOS/*NOS2*) and endothelial (eNOS/*NOS3*). The effects of NO on tumor growth can be dichotomous or context-dependent, depending on the activities and expressions of NOSs, NO concentration and duration of exposure, and cellular sensitivity to NO [21]. Few genetic studies show that polymorphisms in *NOSs* gene are associated with susceptibility of prostate cancer risk and its increased expression is correlated with decreased survival in patients or its advanced progression [22–25]. Metastatic prostate cancer tissues exhibit upregulation of eNOS [26]. Our previous study shows that eNOS exhibits a significant upregulation in clinical CRPC tissues and several in vitro and in vivo models of CRPC, and increased NO production can contribute to the antiandrogen resistance in prostate cancer cells via its suppression of AR activity [27]. All these studies suggest that aberrant eNOS-NO signaling could play a crucial role in the progression of advanced prostate cancer.

The role of NOS-NO signaling in PCSCs still remains unexplored so far. The present study aimed to elucidate the significance of increased eNOS expression and enhanced NO production in the growth regulation of PCSCs and also to characterize the regulatory factors involved in its upregulation and its downstream effectors. Our study showed that enhanced endogenous eNOS expression and increased intracellular NO production could promote the growth of PCSCs and also potentiate the advanced growth of CRPC and metastasis via the activation of the downstream sGC-cGMP-PKG signaling pathway. Our findings also revealed that the eNOS-NO signaling axis activated in PCSCs was co-regulated by the nuclear receptor ERRα and oncogenic transcription factor ERG.

## Methods

### Cell lines

A panel of prostate cancer cell lines, including LNCaP and its antiandrogen-resistant subline LNCaP-BC32, VCaP, 22Rv1, DU145 and PC-3 M, were used in this study. LNCaP, 22Rv1 and DU145 were obtained from ATCC (Manassas, VA); VCaP was provided by Dr. K. Pienta and metastatic PC-3 M was provided by Dr. I. Fidler; LNCaP-BC32 was established previously [27]. LNCaP-ERRα- and DU145-ERRα-transduced cells were generated previously [28, 29]. For conventional adherent 2D culture, LNCaP and 22Rv1 cells were grown in RPMI-1640 medium (ATCC, #30-2001), VCaP cells in DMEM medium (Gibco, #31885023) and DU145 in MEM medium (Gibco, #41500034). The complete growth media were supplemented with 10% FBS (Gibco, #10270106) and 1% penicillin–streptomycin mixture. LNCaP-BC32 cells were maintained in RPMI-1640 complete medium supplemented with 32 μM bicalutamide (Hangzhou Heta Pharm & Chem Co.).

### Non-adherent 3D culture

PCSC-enriched spheroids derived from different prostate cancer cell lines were grown using an agar-based non-adherent 3D culture method as established previously [6]. Briefly, single-cell suspensions were prepared from monolayer cell cultures, suspended in defined serum-free medium [GlutaMax™ DMEM/F12 medium supplemented with 20 ng/mL EGF, 20 ng/mL basic FGF, 4 μg/mL insulin, 1 × B-27 supplement, 1% KnockOut serum replacement and 1% penicillin–streptomycin], seeded onto agar-coated plates and allowed to grow for 1–3 weeks to spheroids, with fresh medium replenished every 3–4 days. Spheroids were collected by either gravitational settlement or filtration using 80-μm cell strainers. Spheroid formation capacity was determined by counting the numbers of spheroids grown from a seeding density of 500 or 1000 cells/6-well plate. For detection and visualization of PCSCs, we used a reporter system SORE6-GFP, which contains a tandem repeat (6 ×) of a composite OCT4/SOX2 response element (SORE6) derived from *NANOG* promoter to drive the reporter GFP, to detect and visualize the PCSCs [6, 30]. Prostate cancer cells were transduced with the lentiviral-based SORE6-GFP reporter. To determine the stemness status or differentiation capability of PCSCs, procedure was performed as follows: (i) single-cell suspensions were prepared from spheroids derived from DU145-SORE6-GFP-transduced cells and re-adherent cultured in complete growth DMEM for 48 h; (ii) SORE6^+^ and SORE6^−^ cells were cytometry-sorted from LNCaP-SORE6-GFP cells cultured under adherent 2D culture condition and separately seeded at low cell density followed by 2D culture for 48 h. Cells with GFP fluorescence were imaged under an inverted fluorescence microscope (Olympus IX83).

### Intracellular NO detection

The intracellular NO production was detected using a cell-permeable NO fluorescent indicator 4-amino-5-methylamino-2’,7’-difluorofluorescein diacetate (DAF-FM-DA) following procedure as described previously [27]. Briefly, adherent 2D culture cells or 3D culture spheroids were incubated in complete media with 5 μM DAF-FM-DA for 20 min at 37 °C. After brief washes with PBS, the indicator-loaded cells were incubated in fresh indicator-free complete media in CO_2_ incubator for another 20 min and then were imaged for intracellular fluorescence signal with a confocal microscope (Olympus FV1200). For NO detection in LNCaP-BC32 cells upon treatments with NOS inhibitors or NOS substrate, LNCaP-BC32 cells were treated with either NOS inhibitor L-NAME (100 μM), L-arginine (500 μM) or vehicle DMSO for 72 h followed by FACS cell sorting of CD133^+^/CD44^+^ cell populations for DAF-FM-DA fluorescence detection of intracellular NO production as described above.

### In vivo tumorigenicity and metastasis analyses

(a) In vivo tumorigenicity analysis. Single-cell suspensions were prepared from either adherent 2D culture or 3D culture spheroids. Suspended cells (1 × 10^4^ cells suspended in 100 μl 1:1 PBS–Matrigel mixture) were injected subcutaneously into the flanks of intact male SCID mice, followed by in vivo growth for 7–8 weeks as described previously [31]. At 8th week, some mice bearing tumors were orchiectomized for development of castration-resistant tumors following procedure as described previously [32]. A patient-derived xenograft (PDX) model of primary androgen-sensitive prostate cancer CWR22 was also used in this study [33]. Xenograft tumors were finely minced and digested with collagenase (Stemcell Technologies). Suspended CWR22 cells (1 × 10^6^ cells suspended in 100 μl Matrigel (Corning) were injected into the flanks of male NSG mice. When tumor sizes reached 0.9 cm^3^ (about 6 weeks post-injection), some tumor-bearing mice were castrated to allow development of castration-resistance as the CWR22-CRPC tumors. Castration relapse tumors were developed at about 2–3 weeks post-castration. Xenograft tumors were harvested for immunohistochemistry or molecular expression analysis. (b) In vivo metastasis analysis. A luciferase-based bioluminescence in vivo imaging method was used to detect in vivo tumor growth and metastasis as described previously [34]. Briefly, pLenti6-eNOS/sheNOS/vector-infected and pLenti-luciferase-labeled PC-3 M cells were inoculated into the dorsal prostate of anesthetized intact male SCID mice, followed by tumor growth for 6 weeks. At 4th–5th weeks, tumor-bearing mice received intraperitoneal injection of D-Luciferin (150 μg or 0.15 mg/g body weight) followed by in vivo bioluminescence imaging for detection of in vivo tumor growth and distal metastasis (Bruker In-Vivo Xtreme Imaging System). At 6th week, tumors and enlarged para-aortic lymph nodes were excised for histopathological examination.

### In vitro growth analyses

(a) Cell viability assay. Single-cell suspensions were prepared from monolayer cell cultures and seeded on 96-well plates at a density of 1 × 10^4^ cells/well. After 24 h, cultured cells were treated with different drugs (targeting different regulators in eNOS-sGC-PKG pathway, ERRα or ERG) or control vehicle for 48–72 h. The viable cells were determined by a colorimetric cell viability assay (Cell Counting Kit-8, Dojindo Molecular Technologies) as described previously [18]. (b) Wound healing assay. Cell migration capacity of PC-3 M-eNOS/PC-3 M-sh-eNOS/PC-3 M-vector-transduced cells were evaluated by wound healing assay followed procedure as described previously [35]. Briefly, cells were seeded onto poly-L-lysine-coated 6-well plates to grow to confluent monolayers for 24 h. Monolayers were starved in serum-free medium for 24 h before making straight scratches using 200-μl pipette tips. After washing with medium to remove cell debris, wounded monolayers were incubated in medium with 1% FBS to minimize cell proliferation. The wound gaps were photographed using a phrase-contrast microscope at regular intervals between 0 and 41 h, and the area of scratches was measured using ImageJ software (NIH, Bethesda). Assays were repeated at least in three independent triplicates.

### Molecular biology and immunoblot analyses

(a) RT-qPCR analysis. Total RNA was extracted from adherent 2D culture cells, spheroids or frozen tumor tissues stored in RNA*later* (Thermo Fisher Scientific) using TRIzol reagent (Molecular Research Center), followed by reverse transcriptase-based cDNA synthesis with genomic DNA elimination (PrimeScript RT reagent kit with gDNA eraser, TaKaRa). A SYBR green-based qPCR assay was performed following procedure as described previously in a real-time PCR system [27]. Relative mRNA expression levels of target genes were determined by the comparative 2^−ΔΔCT^ method and normalized to β-actin (*ACTB*). Information on the primer sequences is listed in Additional file 1: Table S1. (b) Immunoblot analysis. Total cellular proteins were extracted from 2D-cultured cells and 3D-cultured spheroids using ice-cooled RIPA lysis buffer. An enhanced chemiluminescence method was used for immunoblotting detection following procedures as described previously [27] using primary antibodies as follows: eNOS, ERRα, β-actin (cell signaling) and ERG (Abcam). (c) Plasmid construction and lentiviral transduction. Expression plasmid pcDNA3-eNOS-GFP was obtained from Addgene plasmid #22444 [36]. Full-length cDNA of *NOS3* was subcloned into pLenti6 as pLenti6-eNOS for lentiviral transduction. shRNA oligonucleotides targeting eNOS or scramble control (sequence information is listed in Additional file 1: Table S2) were synthesized and inserted into cloning vector pLKO.1-TRC for knockdown experiments obtained from Addgene plasmid #10878 [37]. For lentivirus packaging, subconfluent 293 T cells were transfected with lentivirus packaging plasmid psPAX2 (Addgene plasmid #12260; from Didier Trono), envelope expression plasmid pMD2.G (Addgene plasmid #12259; from Didier Trono) and lentiviral transgene plasmid using jetPRIME transfection reagent (Polyplus). After 48- or 72-h transfection, lentivirus-containing medium was collected, micro-syringe-filtered (0.45 μm) and aliquoted at − 80 °C before use. For generation of stable expression clones, cells were infected with diluted lentivirus-containing medium supplemented with infection regent polybrene (10 μg/ml) for 6 h and recovered in complete medium for another 24 h, followed by antibiotic selection for 1 week and immunoblot validation.

### FACS and MACS cell sorting

(a) Fluorescence-activated cell sorting (FACS). Viable suspended cells (1 × 10^7^) were prepared from either adherent 2D culture or 3D culture spheroids, pre-blocked with PBS containing 0.5% BSA and incubated with 1:11 diluted fluorochrome-conjugated antibodies, CD44-FITC or CD133-APC (Miltenyi Biotec) for 10 min in dark at 2–8 °C. The antibody-labeled cells were washed, re-suspended in PBS and analyzed on a flow cytometer (BD LSRFortessa Cell Analyzer). CD133^+^ cell populations were also sorted out from primary culture prostate cancer cells by FACS. SORE6^+^ and SORE6^−^ cell populations were isolated from adherent 2D culture DU145-SORE6-GFP and LNCaP-SORE6-GFP cells by FACS. (b) Magnetic-activated cell sorting (MACS). Suspended cells (1 × 10^7^) were prepared from LNCaP-BC32 cells, pre-blocked with PBS with 0.5% BSA and incubated with anti-CD44 or anti-CD133-conjugated microbeads for 15 min in dark at 2–8 °C, followed by magnetic separation (MiniMACS Separator, Miltenyi Biotec). FACS- or MACS-sorted cells were analyzed by 3D culture spheroid formation assay or gene expression analysis for stemness markers.

### cGMP measurement

The cellular cGMP levels in either 2D culture cells or 3D culture spheroids were measured by a colorimetric direct competitive anti-cGMP immunoassay (cGMP Direct Immunoassay Kit, Abcam ab65356) according to the manufacturer’s instructions. Briefly, adherent 2D culture cells and 3D culture spheroids were freshly prepared and centrifuged, and incubated with 0.1 M HCl for 10 min on ice. After incubation, samples were centrifuged and supernatants were collected. The cGMP samples in supernatant were acetylated and assayed by direct competition with cGMP-HRP conjugates binding to the Protein G-coated on the plates, followed by absorbance measurement at OD_450nm_. The cGMP concentration in samples, which is inversely proportional to OD_450nm_, was calculated according to the standard curve.

### Statistical analysis

Statistical analysis of difference for continuous variables data was performed by unpaired Student’s *t* test or one-way analysis of variance (ANOVA) using SPSS statistics software with *P* values < 0.05 considered significant. Log-rank test was used for survival analysis.

## Results

### eNOS exhibits an increased expression in metastatic CRPC

**Fig. 1:**
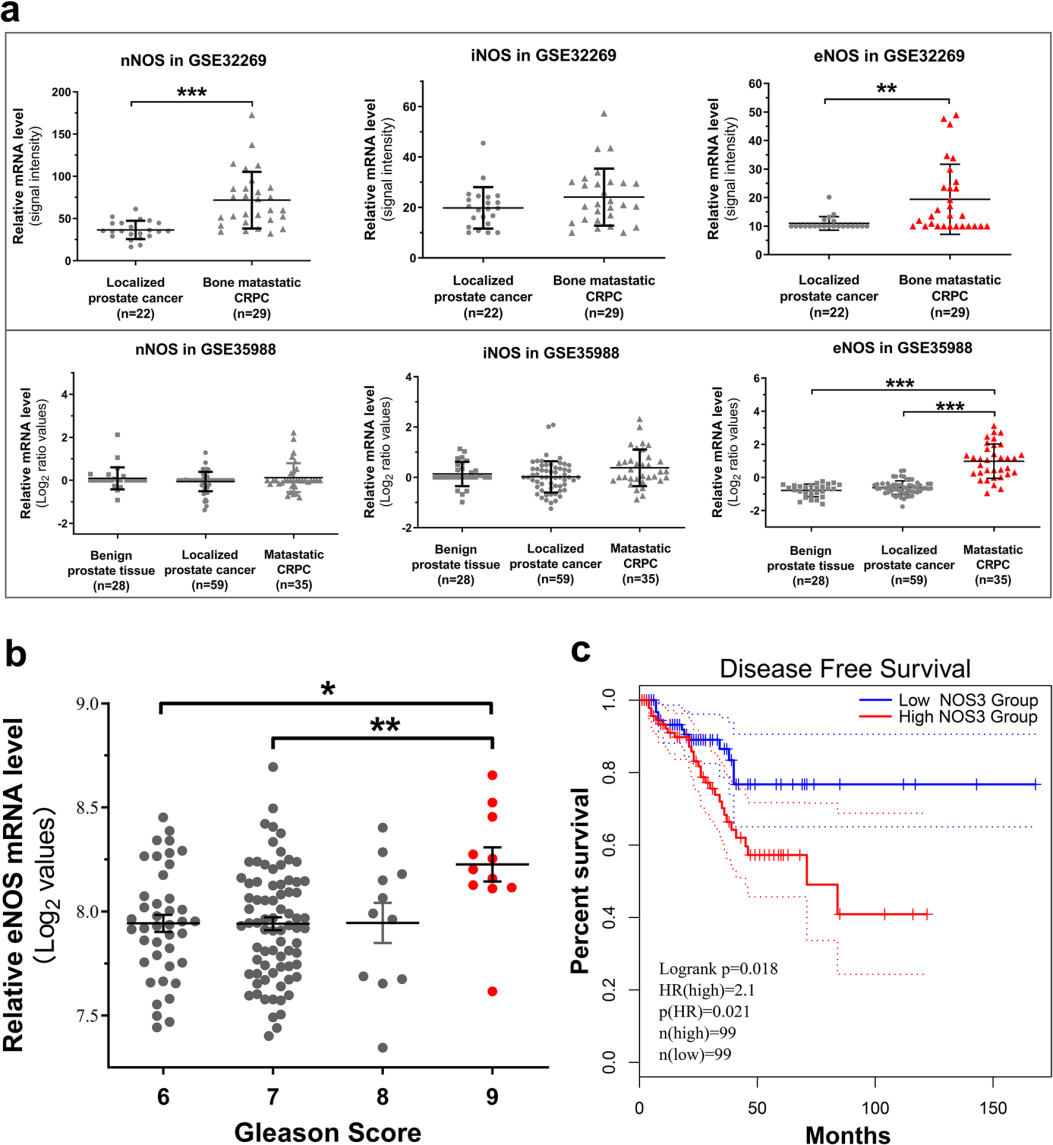
Increased eNOS expression in high-grade prostate cancer and metastatic CRPC. **a** Comparison of mRNA expressions of three NOS isoforms showed that eNOS exhibited a consistent elevated expression pattern in metastatic CRPC tissues as compared to that in benign hyperplastic prostates and localized prostate cancer tissues, as revealed by two microarray expression datasets (GSE32269, GSE35988). **b** eNOS displayed statistical higher mRNA expression in high Gleason score prostate cancers, as revealed by a microarray expression dataset GSE21032. **c** Kaplan–Meier survival curves of cohort from TCGA using the GEPIA2 analysis tool revealed that prostate cancer patients with high eNOS expression level (top 20%) would have a significant shorter disease-free overall survival than patients with low eNOS expression level (bottom 20%). *P < 0.05, **P < 0.01, ***P < 0.001

Previously, we demonstrate that eNOS exhibits an increased immunoexpression pattern in both high-grade hormone-naïve and hormone-refractory (castration-failed and castration-plus-flutamide-failed) prostate cancer samples and also activated eNOS-NO signaling can function to promote the antiandrogen-resistant growth of prostate cancer cells via a mechanism of NO-mediated suppression of AR activity [27]. Here, we continued to explore the expression profile of eNOS in CRPC. Analysis of gene expression profiles in two study cohorts of CRPC available from Gene Expression Omnibus databases GSE35988 [38] and GSE32269 [39] showed that among the three NOS isoforms, eNOS exhibited a significant upregulation in advanced clinical metastatic CRPC samples as compared to benign hyperplastic prostates and localized hormone-naïve prostate cancer samples (Fig. 1a). This expression pattern of eNOS was also confirmed in three used prostate cancer cell lines that only eNOS transcripts but not nNOS and iNOS were detected in LNCaP, DU145 and VCaP cells (Additional file 1: Fig. S1). Analysis of a dataset GSE21032 [40] of gene expression microarray performed in clinical prostate cancer tissues further confirmed that eNOS exhibited significant higher expression levels in higher Gleason score (GS) tumor samples as compared to tumors with lower GS (Fig. 1b). Finally, analysis of TCGA datasets of primary prostate cancer using the GEPIA2 online tool [41] revealed that patient group with high eNOS expression was associated with shorter survival (Fig. 1c). Together, these expression profiles results suggest that eNOS would be a poor prognostic biomarker for prostate cancer and also implicate a positive role in CRPC.

### CRPC models contain higher PCSC populations with enhanced eNOS expression and activity

**Fig. 2:**
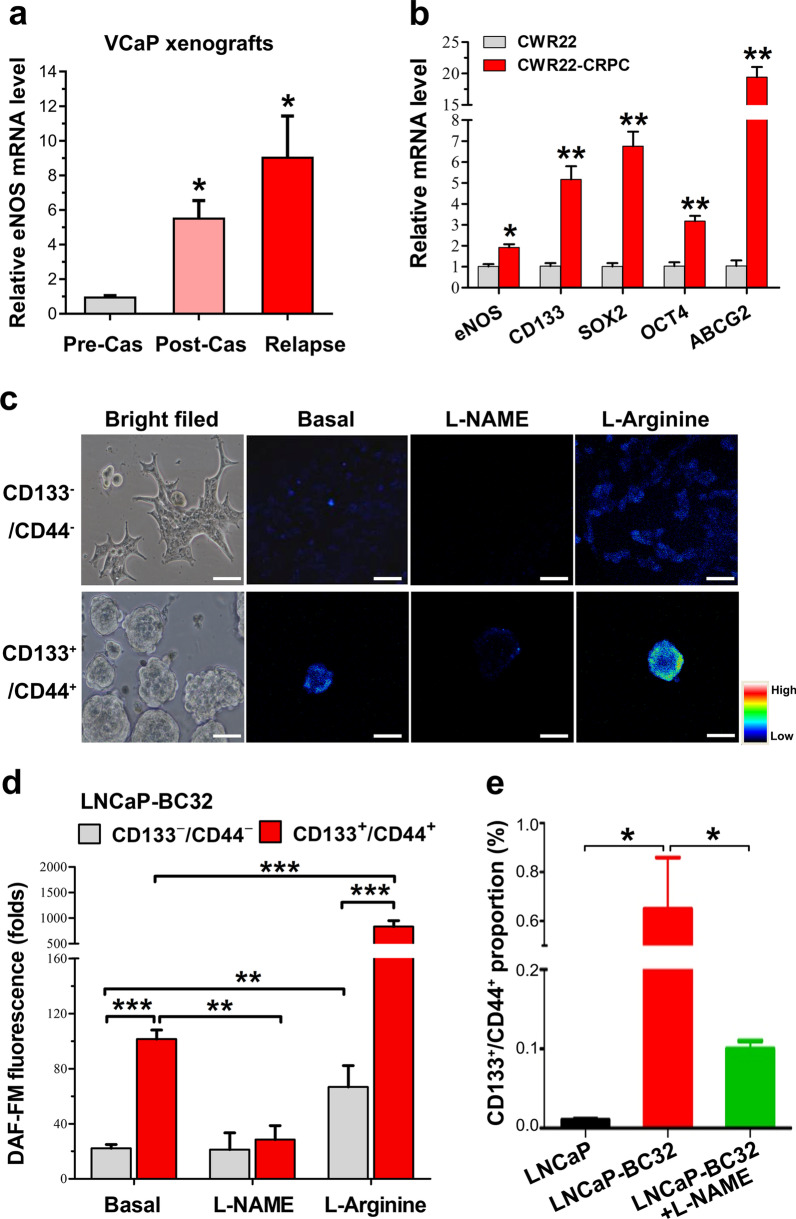
CRPC models contain more PCSC populations with enhanced eNOS expression and higher intracellular NO levels. **a**, **b** Androgen-sensitive and castration relapse VCaP and CWR22 xenografts. q-PCR analysis showed that eNOS exhibited a progressive increase in mRNA levels in xenograft tumors at 4 days (Post-Cas) and 2 months post-castration (Relapse) as compared to tumors before host castration. Results also revealed that the castration relapse CWR22-CRPC xenograft tumors expressed significant higher levels of multiple PCSC-associated biomarkers. **c**, **d** Microscopic detection of intracellular NO by NO fluorescent probe DAF-FM in bicalutamide-resistant LNCaP-BC32 cells-derived and FACS-sorted CD133^−^/CD44^−^ and CD133^+^/CD44^+^ cell populations, grown under either adherent 2D culture or non-adherent 3D culture (spheroids) condition and upon treatments with NOS inhibitor (L-NAME, 100 μM) or substrate (L-Arginine, 0.5 mM). **c** Representative micrographs show the intracellular NO-activated DAF-FM signals. Bars: 50 μm. **d** Semiquantitative analysis of NO levels (DAF-FM fluorescence signals). Results showed that 3D culture spheroids (derived from CD133^+^/CD44^+^ cells) displayed higher intense basal NO signals as compared to adherent 2D culture CD133^−^/CD44^−^ cells without treatment with L-NAME or L-Arginine. Their NO levels as detected in both 3D culture spheroids and adherent 2D culture cells were significantly reduced or abolished upon treatment with L-NAME but significantly intensified upon L-Arginine treatment. **e** FACS CD133-CD44 sorting of LNCaP-BC and LNCaP cells showed that the LNCaP-BC32 cells contained more CD133^+^-CD44^+^ subpopulation than their parental LNCaP cells. The CD133^+^-CD44^+^ subpopulation was significantly reduced/lessened upon treatment with L-NAME (100 μM). Results repeated at least three times are expressed as mean ± SD. *P < 0.05, **P < 0.01, ***P < 0.001

To validate the upregulation of eNOS as shown in clinical metastatic CRPC and determine its role in CRPC, we next examined its expression pattern in two xenograft models of CRPC, based on the castration relapse growth of VCaP cells [32] and patient-derived xenograft (PDX)-derived CWR22 xenograft (Additional file 1: Fig. S2a). Results showed that eNOS displayed a significant increase in both mRNA and protein levels in xenograft tumors at 4 days post-castration, with further elevation in castration relapse tumors developed at 2 months post-castration (Fig. 2a, Additional file 1: Fig. S2b). Previously, we have demonstrated that castration relapse VCaP-CRPC xenograft tumors contain more population of PCSCs [42]. To determine whether eNOS signaling and PCSCs would play roles in the progression of CRPC, we examined the expression profiles of eNOS and CSCs-associated markers in CRPC xenografts. Results showed that CRPC xenografts exhibited remarkable higher mRNA levels of eNOS and also multiple PCSC-associated markers [*PROM1* (CD133), *SOX2*, *POU5F1* (OCT4) and *ABCG2*] (Fig. 2a, b), suggesting that upregulation of eNOS would be accompanied with the enrichment of PCSC populations during CRPC progression. Microscopic detection of intracellular NO levels by NO indicator DAF-FM revealed that the sorted CD133^+^/CD44^+^ LNCaP-BC32 cells exhibited significantly higher NO level (~ 5 folds) than CD133^−^/CD44^−^ cells (Fig. 2c, d). Treatment with NOS inhibitor L-NAME could eliminate completely the NO molecules in CD133^+^/CD44^+^ cells to weak level as in CD133^−^/CD44^−^ cells. Conversely, supply of NOS substrate L-arginine could elevate the NO signals in both CD133^+^/CD44^+^ and CD133^−^/CD44^−^ populations, with significant higher signal in CD133^+^/CD44^+^ cells, suggesting an activation of eNOS enzyme activity in sorted PCSCs. Additionally, FACS analysis of CD133^+^/CD44^+^ cell proportions in bicalutamide-resistant LNCaP-BC32 subline showed that the LNCaP-BC32 cells contained more subpopulation of CD133^+^/CD44^+^ cells (PCSCs) as compared to their parental LNCaP cells (Fig. 2e, Additional file 1: Fig. S3). Furthermore, treatment with NOS inhibitor L-NAME could significantly reduce the CD133^+^/CD44^+^ proportions in LNCaP-BC cells. Together, these results suggest that CRPC tumors and also antiandrogen-resistant cells contained more populations of PCSCs that showed higher intrinsic eNOS expression and enhanced activity.

### Isolated PCSCs display enhanced eNOS-NO signaling

**Fig. 3:**
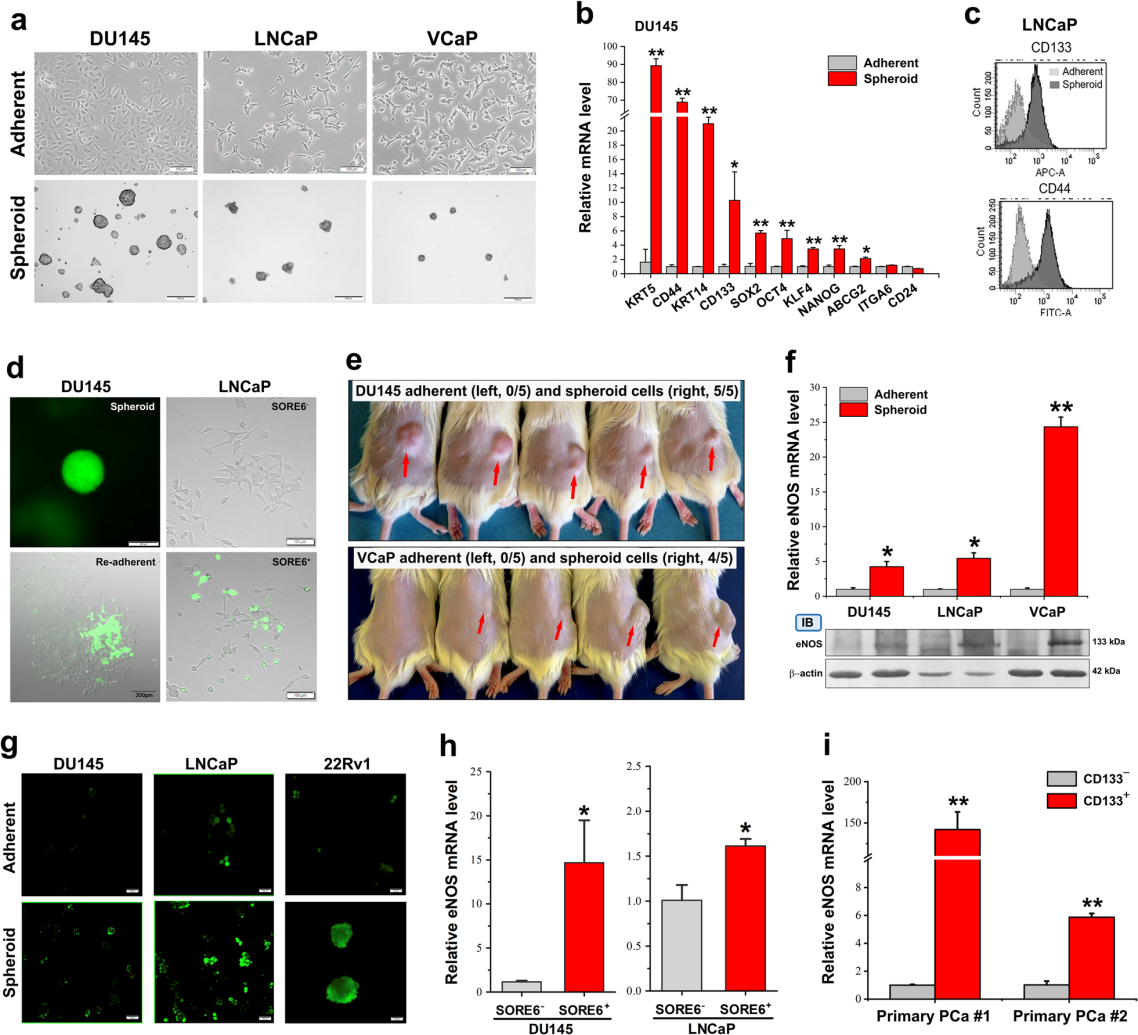
PCSCs exhibit significant activation of eNOS-NO signaling. **a**–**e** Characterization of 3D culture spheroids and SORE6^+^ cells on their PCSC phenotype. **a** Representative micrographs of prostate spheroids. Bars: 2D cultures, 100 μm; spheroids, 200 μm. **b** RT-qPCR analysis of PCSC-associated markers. The spheroids expressed higher levels of multiple PCSC-associated transcription factors (*SOX2, OCT4, KLF4, NANOG*) and membrane antigens (CD44, CD133). **c** FACS CD133-CD44 sorting of LNCaP cells. Results showed that the spheroids contained more subpopulations of CD133^+^/CD44^+^ cells. **d** Fluorescent detection of SORE6-GFP signals in spheroids. Left: The spheroids expressed intense GFP signals. Upon re-adherent differentiation culture for 48 h, 2D cultures still contained small population of SORE6-GFP^+^ cells. Bars: 200 μm. Right: FACS-sorted SORE6^+^- and SORE6^−^-LNCaP cells were re-plated at 2D culture condition for 48 h, and GFP signals were detected in SORE6^+^ LNCaP cells but absent in SORE^−^-LNCaP cells. Bars: 100 μm. **e** In vivo tumorigenicity assay by low-cell-number inoculations (1 × 10^4^ cells per site) of spheroids (inoculation site: right flank, red arrows) versus 2D cultures (left flank). Duration for xenograft tumor growth: DU145 cells for 7 weeks, VCaP cells for 12 weeks. Results showed that almost all spheroids could form xenograft tumors but not 2D cultures. **f** RT-qPCR and immunoblot analyses of eNOS expression. Results showed that the spheroids exhibited significant higher mRNA and protein levels. The immunoblots (IB) were cropped around the bands at 133 kDa and 42 kDa molecular weight markers from different membrane blots. **g** Microscopic detection of intracellular NO using DAF-FM in spheroids versus 2D cultures. Results showed that all spheroids showed more intense NO signal. Bars: 50 μm. **h**, **i** RT-qPCR analysis of FACS-sorted SORE6^+^ cells showed that SORE6^+^ cells expressed significant higher eNOS levels as compared to SORE6^−^ cells. CD133^+^ cells sorted from primary-cultured prostate cancer tissues showed higher eNOS levels as compared to CD133^−^ cells. Results repeated at least three times are expressed as mean ± SD, *P < 0.05, **P < 0.01

Based on the unique growth feature of anchorage-independent growth or anoikis resistance of CSCs, we have developed an improved economical agar-based non-adherent 3D culture method for isolation and enrichment of CSCs derived from different sources [6]. Being prepared by this single-cell 3D culture method, spheroids derived from different prostate cancer cell lines (DU145, LNCaP and VCaP) and CD133^+^-sorted primary-cultured prostate cancer cells displayed significant elevated levels of multiple CSC-associated transcription factors (SOX2, OCT4, KLF4, NANOG) and membrane antigens (CD44, CD133) (Fig. 3a–c, Additional file 1: Fig. S4a-c). Utilizing the CSC-visualizing reporter SORE6-GFP, we also confirmed that the 3D cultured spheroids derived from single-cell suspensions of DU145/LNCaP-SORE6-GFP cells comprised SORE6-responsive cells and possessed the differentiation capacity to SORE6-negative cells, suggesting that the spheroids were enriched of PCSCs (Fig. 3d, Additional file 1: Fig. S4c). Flow cytometry-sorted SORE6^+^ and SORE6^−^ cells derived from their parental LNCaP cells were evaluated for their differentiation capacity by their SORE6-GFP signals. Results showed that the SORE6^+^ cells but not SORE6^−^ cells still maintained responsive SORE6^+^ activation signals, further validating that SORE6^+^ cells were PCSCs (Fig. 3d). These results also suggest that prostate cancer cells under adherent 2D culture contain a small proportion of PCSCs. PCSCs isolated from 3D culture spheroids derived from different prostate cancer cell lines exhibited enhanced in vivo tumorigenicity in host SCID mice by low-cell-number injections (Fig. 3e, Additional file 1: Fig. S4d). These results indicate that the 3D culture spheroids were enriched of PCSCs.

We next sought to determine the expression profile of eNOS in PCSCs-enriched 3D-cultured spheroids. Expression analysis showed that the 3D-cultured spheroids derived from different prostate cancer cell lines expressed significant higher eNOS expression in both mRNA and protein levels, of which levels returned to low levels after re-adherent differentiation culture (Fig. 3f, Additional file 1: Fig. S4c). Microscopic detection of intracellular NO by DAF-DM confirmed that the PCSCs-enriched 3D-cultured spheroids displayed higher intracellular NO level than their corresponding counterparts under adherent 2D culture (Fig. 3g). Moreover, PCSCs isolated by SORE6-GFP reporter-based FACS sorting of different prostate cancer cell lines showed that SORE6^+^ cells expressed significant higher level of eNOS than SORE6^−^ cells (Fig. 3h). Similarly, PCSCs isolated by anti-CD133-based FACS sorting further confirmed that PCSCs derived from primary prostate cancer cultures expressed higher level of eNOS (Fig. 3i). Together, these results indicate that PCSCs isolated from either prostate cancer cell lines or primary prostate tumor cultures exhibited enhanced eNOS-NO signaling or activity.

### Enhanced eNOS expression can promote in vivo tumor growth and metastasis potential of prostate cancer cells

**Fig. 4:**
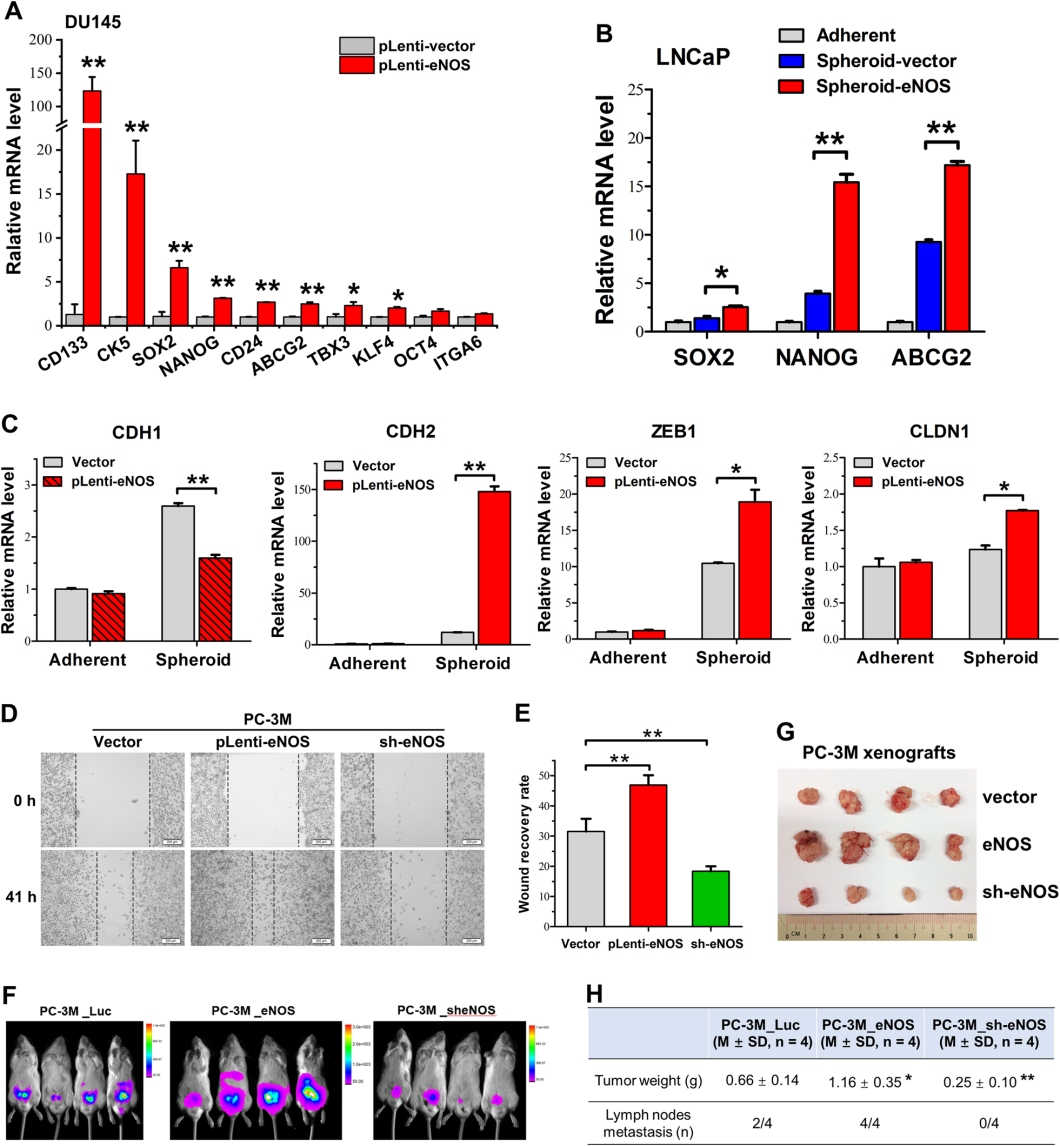
eNOS can promote EMT of PCSCs and in vivo prostate tumor growth and metastasis. **a**, **b** RT-qPCR analysis of stemness-associated genes in adherent 2D culture cells and 3D spheroids with eNOS transgene overexpression. Results showed that eNOS overexpression induced significant upregulation of stemness genes in adherent 2D culture DU145 cells and also 3D culture LNCaP spheroids. **c** RT-qPCR analysis of four EMT-associated markers [mesenchymal markers: *CHD2* (N-cadherin), EMT-inducing factors: transcription factor *ZEB1* and *CLDN1* (claudin-1); epithelial marker: *CDH1* (E-cadherin], in LNCaP-eNOS cells. Results showed that eNOS overexpression could induce significant upregulation of CDH2, ZEB1 and CLDN1 but downregulation of CDH1 in 3D culture spheroids formed by LNCaP-eNOS cells as compared to spheroids formed by the LNCaP vectors cells. **d**, **e** Wound healing assay. **d** Representative images of PC-3 M-vector/-eNOS/-sheNOS-transduced cells taken at 0 h and 41 h time point. Bar: 200 μm. **e** Semiquantitative analysis of wound closure determined by measurement of width of wounds. Results showed that PC-3 M-eNOS cells showed significant higher migration capacity, whereas PC-3 M-sh-eNOS cells showed reduced capacity, as compared to PC-3 M-vector cells. **f** Luciferase-based bioluminescence in vivo imaging. Representative bioluminescence pictures of mice at 5 weeks post-inoculation of PC-3 M-vector/eNOS/sh-eNOS cells. Intense bioluminescence tumor growth signals were detected in the prostates of mice which had received orthotopic inoculation of PC-3 M-eNOS cells. Moderate and very weak bioluminescence signals were detected in mice bearing inoculations of PC-3 M-vector or PC-3 M-sh-eNOS cells, respectively. **g** Photograph shows the dissected prostate tumors formed by the inoculated PC-3 M-vector/eNOS/sh-eNOS cells in mice. Significant larger tumors were formed by PC-3 M-eNOS cells. **h** Table summarizes the tumor weights and detected metastasis to para-aortic lymph nodes in host mice bearing xenografts of PC-3 M-vector/eNOS/sh-eNOS cells. Results repeated at least three times are expressed as mean ± SD, *P < 0.05, **P < 0.01

Previous studies show that androgen deprivation therapy can induce and also promote epithelial–mesenchymal transition (EMT) in prostate cancer, and the EMT process is shown to be closely associated with cancer stemness or PCSC growth [43, 44]. To explore the significance of eNOS-NO signaling in EMT in PCSCs and also prostate cancer metastasis, eNOS was overexpressed in 2D-cultured prostate cancer cells and 3D-cultured spheroids for phenotype evaluation. Results showed that eNOS overexpression could induce remarkable upregulation of multiple CSCs markers (CD133) and stemness-associated genes, especially transcription factors *NANOG* and *SOX2*, in prostate cancer cells under adherent 2D culture (Fig. 4a). Ectopic expression of eNOS in spheroids validated that eNOS-NO signaling could promote stemness features of prostate cancer cells (Fig. 4b). Further analysis showed that LNCaP-derived spheroids exhibited significant higher expression levels of EMT-inducing factors [transcription factor *ZEB1* and *CLDN1* (Claudin-1)] and mesenchymal marker *CDH2* (N-cadherin) as compared to their corresponding cells grown under adherent 2D culture condition, with their further expressions in 3D-cultured spheroids formed by eNOS-overexpressed cells (Fig. 4c). Conversely, overexpression of eNOS could suppress the epithelial marker *CDH1* (E-cadherin) in LNCaP-eNOS cells when grown in non-adherent 3D culture condition. These results suggest that enhancement of eNOS-NO signaling could maintain the stemness of PCSCs and induce the EMT process in PCSCs. In vitro wound healing assay showed that eNOS overexpression could remarkably promote, whereas its knockdown could suppress, the migration capacity of PC-3 M cells, a highly metastatic subline of PC-3 (Fig. 4d, e). In vivo tumorigenicity assay further showed that overexpression of eNOS could promote, whereas its knockdown could inhibit, both the tumorigenicity and also the lymph node metastasis potential of PC-3 M cells grown in intact host mice (Fig. 4f–h). Together, these results suggest that enhancement of eNOS signaling could promote the EMT process in PCSCs, which then enhance tumor growth and metastasis potential of prostate cancer.

### Enhanced eNOS signaling can promote the growth of PCSCs and antiandrogen-resistant prostate cancer cells via an activation of NO-sGC-cGMP-PKG signaling pathway

**Fig. 5:**
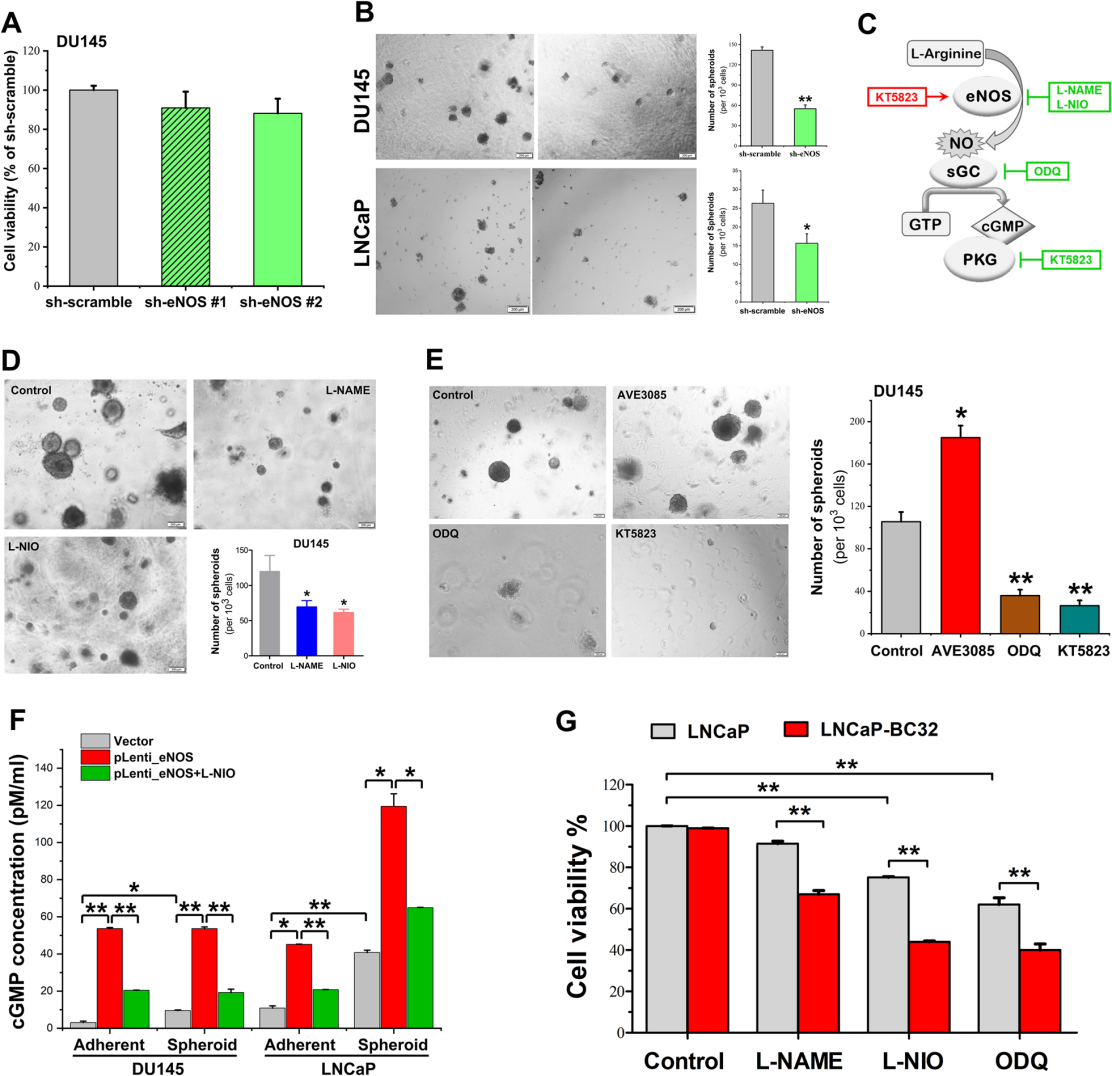
Activation of eNOS-NO signaling and its downstream effectors in PCSCs and antiandrogen-resistant prostate cancer cells. **a** Cell viability assay. DU145-sh-eNOS cells grew at the same proliferation rate as DU145-sh-scramble cells under adherent 2D culture condition, suggesting that knockdown of eNOS induced no significant impact on proliferation of DU145 cells. **b**–**d** 3D culture spheroid formation assay performed on DU145 and LNCaP cells. **b** Knockdown of eNOS by sh-eNOS could significantly suppress the spheroid formation capacity of DU145 and LNCaP cells. **c** Schematic diagram illustrates the actions of different used selective activator and inhibitors of regulators of the eNOS-NO-sGC-cGMP-PKG signaling pathway. **d** Treatment with eNOS inhibitors, L-NAME and L-NIO (100 μM, respectively), could significantly suppress the spheroid formation capacity of DU145 cells. **e** Treatment with an *NOS3* enhancer AVE3085 (5 μM) could significantly enhance the spheroid formation capacity of DU145 cells. However, treatments with selective sGC inhibitor ODQ (20 μM) and PKG inhibitor KT5823 (10 μM) could significantly suppress the spheroid formation capacity of DU145 cells. **f** Measurement of cGMP levels in DU145-eNOS and LNCaP-eNOS-transduced cells by direct immunoassay. Results showed that both DU145-eNOS and LNCaP-eNOS cells contained significant higher cGMP levels than their corresponding parental cells, grown as either adherent 2D culture cells or 3D culture spheroids, and with such increased cGMP levels being suppressed by L-NIO treatment (100 μM). **g** Cell viability assay performed in LNCaP-BC32 cells. Analysis showed that treatments with inhibitors targeting to eNOS (L-NAME, 100 μM; L-NIO, 200 μM) and sGC (ODQ, 20 μM) could induce more growth inhibition on LNCaP-BC32 cells than LNCaP cells. Results repeated at least three times are expressed as mean ± SD, *P < 0.05, **P < 0.01

To evaluate the functional significance of eNOS-NO signaling in the growth regulation of PCSCs, we next determined the growth impact of either knockdown or overexpression of eNOS and also pharmacological suppression of eNOS activity on the non-adherent 3D culture growth capacity (stemness feature) of prostate cancer cells. Studies showed that shRNA-mediated knockdown of eNOS induced no significant growth impact on the adherent 2D cultures of DU145 (Fig. 5a). However, knockdown of eNOS could remarkably suppress the 3D-cutlure spheroid formation capacity of DU145 and LNCaP cells (Fig. 5b). Furthermore, suppression of eNOS activity by NOS inhibitors, L-NAME and L-NIO, could also significantly suppress the 3D culture spheroid formation capacity of prostate cancer cells (Fig. 5c, d). These results suggest that activation of eNOS-NO signaling could function to promote the in vitro growth of PCSCs regardless of their AR expression status. It is well characterized that NOS-NO signaling or NO can primarily lead to the activation of soluble guanylate cyclase (sGC) to produce the second messenger cyclic guanosine monophosphate (cGMP) and then activate one of its main target effectors cGMP-dependent protein kinase (PKG), which in turn regulates diverse activities involved in multiple cellular and physiological processes [45]. We next employed multiple selective activator and inhibitors of eNOS-sGC-PKG signaling pathway to confirm whether the activation or enhancement of eNOS-NO-sGC-PKG signaling pathway would be involved in the growth regulation of PCSCs and CRPC (Fig. 5c). In vitro analyses showed that treatment with AVE3085, a potent eNOS enhancer, could significantly promote the 3D culture spheroid formation capacity of DU145 cells in both spheroid numbers and sizes (Fig. 5e). Conversely, treatments with selective inhibitors of sGC (ODQ) and PKG (KT5823) could significantly suppress the spheroid formation capacity of DU145 and 22Rv1 cells but not affect their growth under adherent 2D culture (Fig. 5e, Additional file 1: Fig. S5a, b). We next analyzed the cGMP levels in prostate cancer cells grown under adherent 2D culture or non-adherent 3D culture conditions. Immunoassay results showed that both DU145- and LNCaP-derived 3D culture spheroids contained higher cGMP levels than their corresponding adherent 2D culture cells; and their cGMP levels could be significantly elevated by overexpression of eNOS in cells grown under adherent 2D- or non-adherent 3D culture conditions and with the elevated levels attenuated by L-NIO treatment (Fig. 5f). Further study performed in antiandrogen-resistant LNCaP-BC32 cells showed that treatments with eNOS inhibitors (L-NAME and L-NIO) and sGC inhibitor (ODQ) could exert further growth inhibition on LNCaP-BC32 cells as compared to their parental hormone-sensitive LNCaP cells (Fig. 5g). These results further expand on our previous findings that activation of eNOS-NO signaling can contribute to the antiandrogen resistance in prostate cancer cells [27] by promotion of sGC-PKG-dependent growth of PCSCs. Taken together, our results suggest that eNOS could function to promote the growth of PCSCs and also CRPC via the activation of downstream effector NO-sGC-cGMP-PKG signaling pathway.

### ERRα and ERG can directly co-activate eNOS signaling in prostate cancer cells

**Fig. 6:**
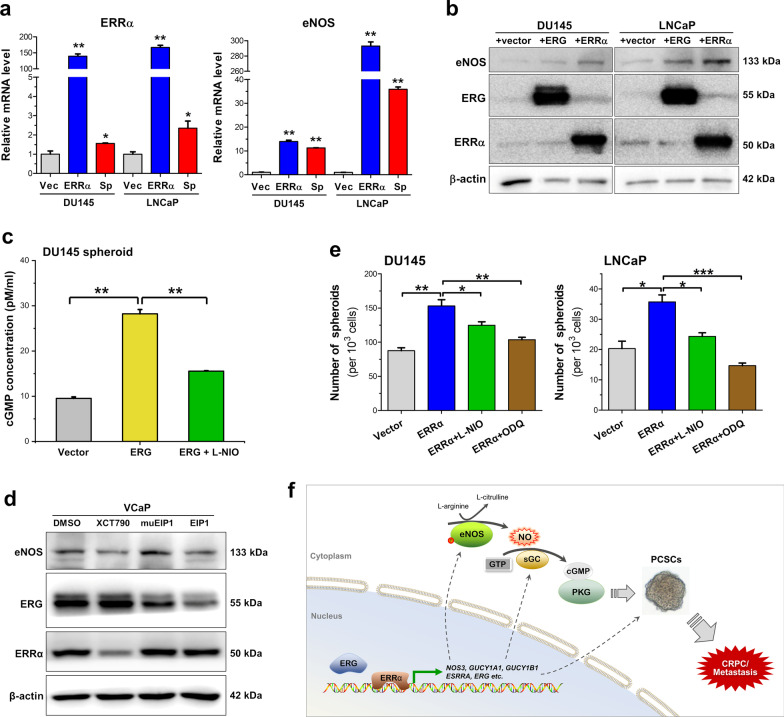
ERRα and ERG can act to co-activate the eNOS-NO signaling in prostate cancer cells. **a** RT-qPCR analysis of ERRα and eNOS expression in prostate 3D culture spheroids versus adherent 2D culture cells. Left: spheroids (Sp) grown from DU145 or LNCaP cells exhibited higher ERRα expression levels as compared to their corresponding 2D cultures. Right: spheroids exhibited significant higher eNOS expression as compared to their parental cells. Overexpression of ERRα in 2D-cutured cells could significantly upregulate eNOS expression (blue bars). **b** Immunoblot analysis of eNOS, ERG and ERRα in DU145 and LNCaP cells. The blots were cropped around the bands at 133, 55, 50 and 42 kDa molecular weight markers from different membrane blots. Results showed that transient overexpression of either ERG or ERRα could significantly enhance protein expression of eNOS. **c** Measurement of cGMP levels in 3D culture spheroids derived from DU145-ERG-transduced cells. Analysis showed that DU145-ERG spheroids contained higher cGMP concentration levels than DU145-vector spheroids, and with such increased cGMP levels being eliminated by L-NIO treatment. **d** Immunoblot analysis of eNOS, ERG and ERRα in VCaP cells upon treatments with ERRα inverse agonist XCT790 (5 μM), ERG inhibitory peptide EIP1 (50 μM) or its negative control muEIP1 (50 μM). The blots were cropped around the bands at 133, 55, 50 and 42 kDa molecular weight markers from different membrane blots. Results showed that inhibition of ERRα by XCT790 and also ERG by EIP1 could moderately reduce the eNOS levels in VCaP cells. **e** 3D culture spheroid formation assay performed on ERRα-overexpressed prostate cancer cells. Results showed that overexpression of ERRα could significantly promote the spheroid formation capacities of both DU145 and LNCaP cells. However, their spheroid formation capacities could be weakened by treatments with inhibitors of NOS (L-NIO, 100 μM) or sGC (ODQ, 20 μM). Results repeated at least three times are expressed as mean ± SD, *P < 0.05, **P < 0.01. **f** Schematic diagram illustrates the contribution of regulatory loop between ERRα and ERG in the activation of eNOS-NO-sGC-cGMP-PKG signaling pathway in the regulation of PCSCs and CRPC

Previously, it has been characterized that nuclear receptor ERRα can directly transactivate the *NOS3* gene promoter and upregulate the eNOS expression in bovine pulmonary artery endothelial cells [46]. Recently, we show that ERRα can directly transactivate the *TMPRSS2:ERG* fusion gene to drive the expression of oncogenic transcription factor ERG in prostate cancer cells regardless of their AR expression status, and both ERRα and ERG can form a synergistic reciprocal regulatory loop to promote the malignant growth and metastasis potential of prostate cancer cells [29]. Moreover, a recent study shows that ERG can activate cGMP expression in prostate cancer cells [47]. Our present expression analysis revealed that 3D culture PCSCs-enriched spheroids derived from DU145 and LNCaP cells exhibited significant higher expression of ERRα as compared to their counterpart cells grown under adherent 2D culture condition (Fig. 6a). Based on this, we hypothesize that ERRα and ERG could act together as the up-stream regulators of eNOS signaling in PCSCs. Our results showed that overexpression of either ERRα or ERG could induce significant elevation of eNOS at both mRNA and protein levels in DU145 and LNCaP cells (Fig. 6a, b). Assay of cGMP levels in DU145-derived 3D culture spheroids showed that ERG overexpression could induce a significant increase in cGMP level in DU145-derived spheroids and with the levels significantly reduced by eNOS inhibitor L-NIO (Fig. 6c). Furthermore, treatments with inhibitors of ERRα (XCT790) and ERG (EIP1) could reduce the protein levels of eNOS in VCaP cells (Fig. 6d), suggesting that both ERRα and ERG could act as the upstream regulators of eNOS in prostate cancer cells and PCSCs. Spheroid formation assay showed that both DU145-ERRα- and LNCaP-ERRα-transduced cells exhibited significant higher 3D culture spheroid formation capacity, of which could be moderately weakened by NOS inhibitor L-NIO and sGC inhibitor ODQ (Fig. 6e). Together, these results suggest that both ERRα and ERG could act to promote the growth of PCSCs, at least partially, via their activation of eNOS-NO signaling (Fig. 6f).

## Discussion

In the present study, we demonstrated that eNOS exhibited a significant increased expression in high-grade prostate cancer, and also metastatic CRPC, further confirming our previous findings showing its upregulation in CRPC tissues and also an antiandrogen-resistant cell line model [27]. Our further functional analyses also supported that enhanced eNOS expression could promote the in vivo tumorigenicity and potentiate metastasis of prostate cancer cells. Additionally, we also showed that PCSCs, isolated from various in vitro and in vivo CRPC models, displayed a distinct feature of enhanced eNOS expression and higher intracellular NO production as compared to their non-CSC counterparts. These results strongly suggest an integral role of eNOS-NO signaling in PCSCs, which could contribute to the progression of castration-resistance and metastasis of advanced prostate cancer.

We further characterized that the enhanced eNOS-NO signaling could significantly promote the growth of PCSCs regardless of their AR expression status, and also antiandrogen-resistant prostate cancer cells via the activation of downstream NO-sGC-cGMP-PKG signaling pathway, as evidenced by pharmacological inhibition of effectors in this signaling pathway. Moreover, our findings also showed that pharmacological blockage of individual signaling effectors of eNOS-NO-sGC-cGMP-PKG pathway or knockdown of eNOS could impair the stemness (spheroid growth capacity) of PCSCs and also inhibit the in vivo orthotopic tumor growth of prostate cancer cells, suggesting that targeting eNOS-NO-sGC-cGMP-PKG signaling may have therapeutic potential in PCSC targeting and also management of advanced prostate cancer. Accumulating studies show that increased NO production as a result of upregulation of iNOS or eNOS can promote the stemness phenotypes of cancer cells derived from various tumor sources or experimental models, including glioma [48, 49], liver cancer [50], colon cancer and intestinal tumors [51, 52], prostate cancer [47] and bladder cancer [53]. However, unlike the dichotomous effect of NOSs-NO signaling in bulk cancer cells, there are no studies that report the inhibitory effect of endogenous NOS-NO signaling in CSCs. The NO-mediated regulation of CSCs is shown to involve different mechanisms or pathways, including upregulation and protein stabilization of certain stem cells-associated transcription factors (e.g., SOX2) [49, 54], activation of Notch signaling [50] and enhanced expression of Wnt signaling regulator β-catenin and polycomb oncogenic protein Bmi1 [52]. The downstream effectors of activated NO-cGMP-PKG signaling in PSCSs are not characterized yet. Transcriptome sequencing analysis of eNOS-knockdown LNCaP cells identified three marker genes (*HES6, F2RL1* and *KDM3A*) to be significantly regulated by the activated eNOS-NO signaling. Expression analysis confirmed that *HES6* and *F2RL1* showed reduced expressions in LNCaP-sheNOS cells but exhibited significant higher expressions in PSCS-enriched 3D-cultured spheroids as compared to their corresponding adherent 2D-cultured cells. Contrarily, *KDM3A* was repressed by the activated eNOS-NO signaling in PSCS-enriched 3D-cultured spheroids (unpublished data). In all, our present study and also others indicate that activation of NOS-NO signaling or increased intracellular NO production is a common feature of CSCs originated from different cancer types and also its increased level is critical to their growth and maintenance.

The consequent functional impact of enhanced eNOS-NO signaling in PCSCs is still unclear. Previously, we have demonstrated that increased eNOS expression and NO production can contribute to antiandrogen-resistant growth of prostate cancer cells via a mechanism of suppression of AR transactivation and also activation of protein kinase Akt [27]. Our present findings are also supported by another study showing that increased intracellular NO levels can affect the tumor growth of both androgen-dependent and castration-resistant prostate cancer cells through a mechanism of attenuation of AR activity by S-nitrosylation of C601 amino acid residue present at the DNA-binding domain of AR [55]. In fact, we also observe that suppression of AR signaling (reduced KLK2 and KL3 levels) is shown in the 3D culture PCSC-enriched spheroids [6]. Here, we also observed that overexpression of eNOS could significantly upregulate the expression of EMT-associated markers (CDH2, ZEB1 and CLDN1) but downregulate the expression of an epithelial marker CDH1 in PCSCs, suggesting that enhanced eNOS-NO signaling could also promote EMT in PCSCs, likely mediated through a similar but yet-to-be proved mechanism of attenuation of AR activity. It is also shown that androgen deprivation can induce EMT in both normal prostate and prostate cancer [43]. Indeed, accumulating studies suggest that there are cross talks between multiple pathways involved in regulation of both cancer stemness and EMT in the development of CRPC [44]. Based on these, it is believed that enhanced eNOS-NO signaling could lead to inactivation of AR transactivation and induction of EMT in PCSCs via similar mechanisms.

In addition to CSCs, NO derived from other cell types, including tumor-associated macrophages (TAMs), tumor-infiltrating myeloid-derived suppressor cells (MDSCs) and endothelial cells have been demonstrated to be involved in the cancer development [56]. Lung TAMs specifically express elevated iNOS-NO that promotes macrophages survival and infiltration and thus promotes lung carcinogenesis in mouse model [57]. M2 TAMs with low level NO as produced by iNOS are able to protect tumor cells from cisplatin-induced apoptosis via the PKG-dependent CD95-acid sphingomyelinase pathway inhibition [58]. In contrast, high-level NO released from NO donor can inhibit the abundance of M2 macrophages as well as CRPC progression in prostate cancer [59]. MDSC-derived NO production can antagonize the Fc receptor mediated-signal transduction in natural killer (NK) cells and downstream effector functions in vivo, which thus results in tumor immune escape upon monoclonal antibody (mAb) therapy [60]. In the TAMs and MDSCs, iNOS is shown to be the major active NO synthase to produce NO, whereas in the PCSCs, our results showed that eNOS is the only NO synthase activated to generate NO molecules. NO, as synthesized by eNOS and derived from endothelial cells, has been shown to promote the angiogenesis in tumor tissues [61]. However, one recent single-cell sequencing study shows that the proportion of endothelial cells in the prostate tumor tissues is quite low (less than 7% of total cell population), in 6 out of 7 localized prostate tumors obtained by radical prostatectomy [62], and thus their possible contribution in terms of eNOS-NO production to the growth of tumor cells or PCSCs is believed to be minimal. Moreover, emerging evidence demonstrates that PCSCs are resistant to androgen deprivation therapy and their expansion in cell population during the advanced progression would lead to CRPC which exhibits stem-like features [7]. Together, our present results support that the eNOS-NO signaling is activated in PCSCs, and the elevated eNOS expression or NO level in prostate tumor tissues may be mainly generated by the PCSCs or its derived cells.

Here, we also characterized that orphan nuclear receptor ERRα and oncogenic transcription factor ERG could act, respectively, to promote the growth of PCSCs via their likewise induction of eNOS expression and enhanced eNOS-NO signaling in PCSCs. Importantly, our study also showed that treatments with either specific ERRα or ERG inhibitors could suppress the eNOS expressions in prostate cancer cells, suggesting that targeting the upstream regulators of eNOS could also act to suppress the eNOS-NO signaling and also implicate their potential therapeutic application in the management of advanced prostate cancer. Indeed, our previous study shows that ERRα and ERG can regulate each other in a synergistic manner and form a reciprocal regulatory loop to advance the progression of prostate cancer [29]. Our present findings also suggest that ERRα and ERG could form a regulatory loop, similar to their synergistic regulation of *TMPRSS2:ERG* fusion gene, to activate the eNOS-NO signaling in PCSCs (Fig. 6f). Two previous studies show that *NOS3* is a direct target of ERRα in endothelial cells and nuclear receptor transcriptional co-regulator PGC-1α is notably involved in such ERRα-induced eNOS expression in endothelial cells [46, 63]. So far, there is no evidence suggesting that eNOS is a direct target of ERG. Our preliminary reporter gene analysis demonstrated that *NOS3* could not be directly transactivated by ERG in prostate cancer cells. A recent study shows that ERG expressed by the *TMPRSS2:ERG* fusion gene can directly regulate both the α1 and β1 subunits of sGC and contribute to promote the downstream effector cGMP-PKG activity in prostate cancer cells [47]. In all, our findings also implicate that besides direct targeting eNOS and also its downstream NO-mediated signaling effectors (such sGC and PKG), targeting the upstream regulators of eNOS could be an alternative approach to attenuate the activated eNOS-NO signaling in PCSCs in order to suppress their stemness or growth in prostate cancer.

## Conclusions

In summary, the results show that increased eNOS-NO signaling is a distinct phenotype of PCSCs and its enhanced activation could function not only to promote the growth of PCSCs but also to advance the malignant growth of prostate cancer via an activation of downstream NO-sGC-cGMP-PKG signaling. Moreover, ERRα and ERG could form a co-activation loop to upregulate the eNOS-NO signaling in PCSCs. Together, our findings implicate that besides eNOS-NO as potential targets, targeting its upstream regulators (ERRα and ERG) could also be the PCSC-directed therapeutic strategy for the management of advanced prostate cancer, particularly the aggressive cancer carrying with the *TMPRSS2:ERG* fusion gene.

## Supplementary Information

**Additional file 1**. Supplementary Figures S1-S5 and Supplementary Tables S1 and S2.

## Data Availability

The data generated during this study are included in this article [and its supplementary information files]. The datasets analyzed during the current study are available in the GEO repository, https://www.ncbi.nlm.nih.gov/geo/query/acc.cgi?acc=GSE35988 [38]. https://www.ncbi.nlm.nih.gov/geo/query/acc.cgi?acc=GSE32269 [39]. https://www.ncbi.nlm.nih.gov/geo/query/acc.cgi?acc=GSE21032 [40].

## Abbreviations

AR: Androgen receptor
cGMP: Cyclic guanosine monophosphate
CRPC: Castration-resistant prostate cancer
CSCs: Cancer stem-like cells
EIP1: ERG inhibitory peptide 1
EMT: Epithelial–mesenchymal transition
eNOS/NOS3: Endothelial nitric oxide synthase
ERRα: Estrogen-related receptor alpha
FACS: Fluorescence-activated cell sorting
iNOS/NOS2: Inducible nitric oxide synthase
nNOS/NOS1: Neuronal nitric oxide synthase
NO: Nitric oxide
PCSCs: Prostate cancer stem-like cells
PDX: Patient-derived xenograft
PKG: CGMP-dependent protein kinase
sGC: Soluble guanylate cyclase
