# Supplementary material for: Endothelial nitric oxide synthase (eNOS)-NO signaling axis functions to promote the growth of prostate cancer stem-like cells
Source: Stem Cell Res Ther. 2022 May 7;13:188. doi: 10.1186/s13287-022-02864-6 (PMC9080127; doi:10.1186/s13287-022-02864-6)

**Endothelial nitric oxide synthase (eNOS)-NO signaling axis functions to promote the growth of prostate cancer stem-like cells**

Weijie Gao, Yuliang Wang, Shan Yu, Zhu Wang, Taiyang Ma, Andrew Man-Lok Chan, Peter Ka-Fung Chiu, Chi-Fai Ng, Dinglan Wu and Franky Leung Chan

**Supplemental Figures**


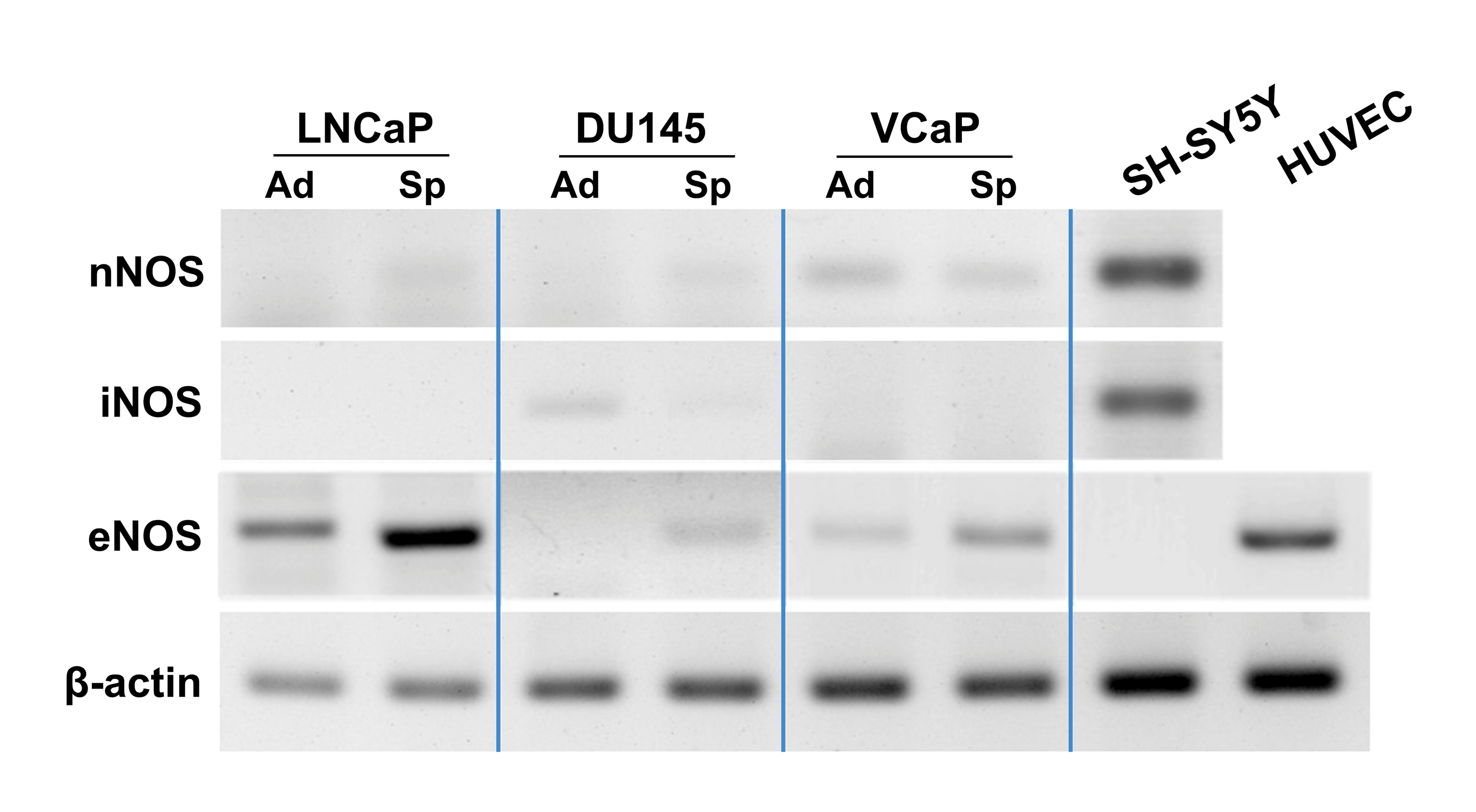


**Fig. S1.** PCSCs-enriched non-adherent 3D-culture spheroids derived from different prostate cancer cell lines display up-regulation of eNOS expression. PCR analysis of eNOS expression in 3D-culture spheroids versus their corresponding adherent 2D-culture prostate cancer cells. Results showed that only eNOS transcripts were detected LNCaP, DU145 and VCaP cells, with particular higher levels detected in their corresponding 3D-culture spheroids. SH-SY5Y cells were used as positive control for nNOS and iNOS, and HUVEC cells for eNOS.


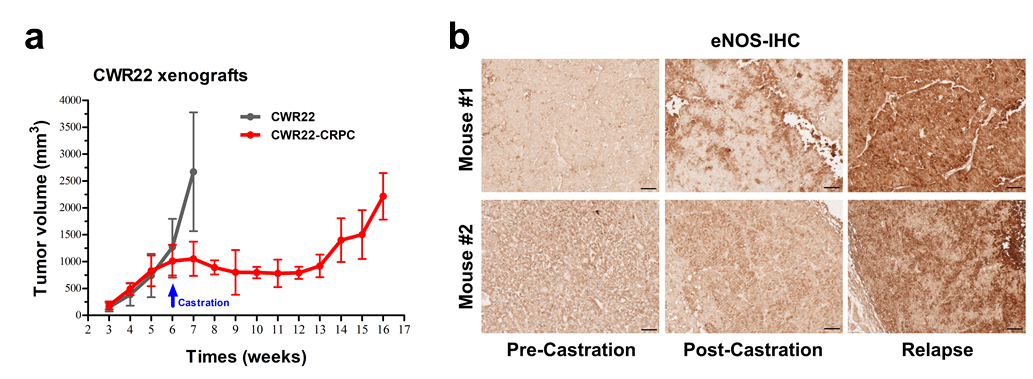


**Fig. S2.** CWR22-CRPC xenograft model exhibits enhanced eNOS expression. **a** Growth curve of CWR22 and CWR22-CRPC xenograft tumors in intact and castrated host SCID mice. Castration was performed on animals at 6-week post-inoculation of CWR22 cells. Relapse growth of tumors was detected in mice at about 2-month post-castration. **b** eNOS immunohistochemistry performed on CWR22 xenograft tumors collected at day when castration was performed (Pre-Castration), 4-day post-castration (Post-Castration) and 16-weeks post-inoculation (Relapse). Intense cytoplasmic eNOS immunosignals were detected in castration-relapse tumors.


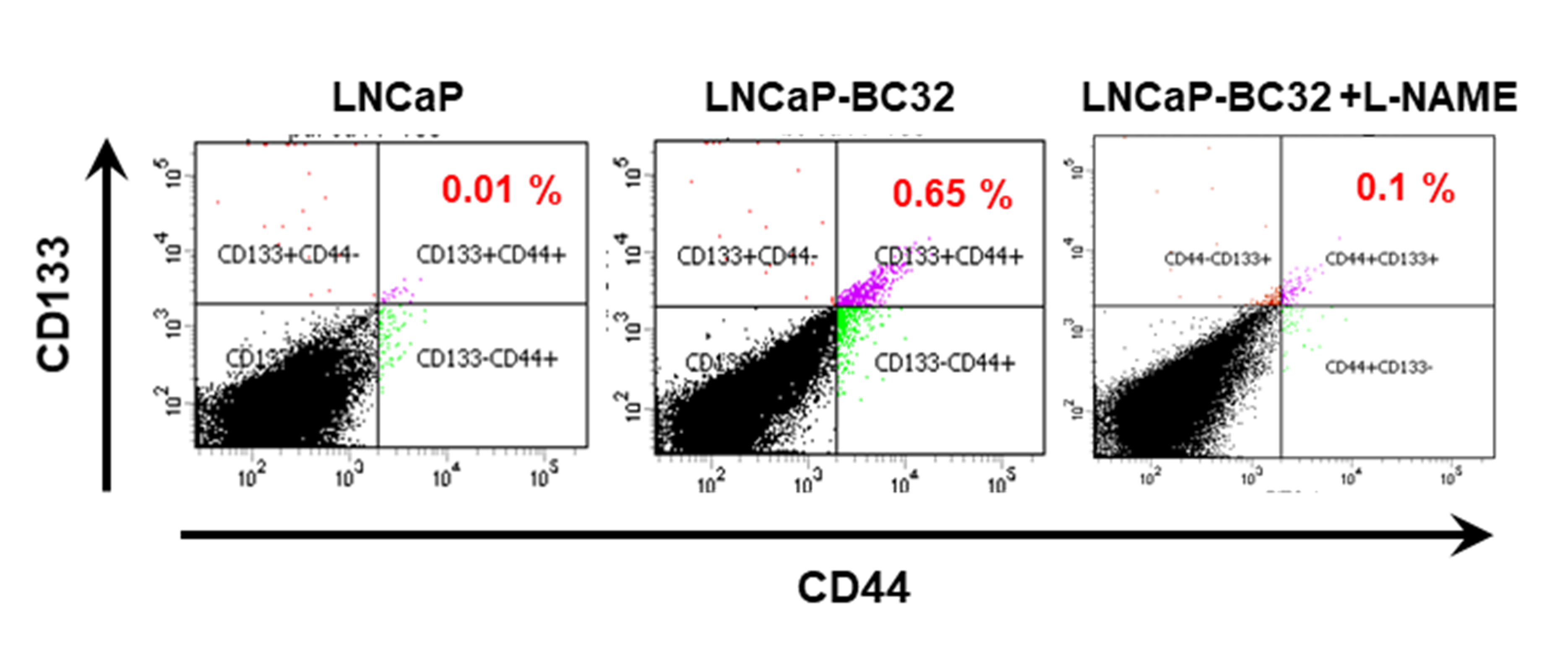


**Fig. S3.** Antiandrogen-resistant LNCaP-BC32 cells contain more population of PCSCs. FACS analysis of CD133^+^/CD44^+^ populations in LNCaP and LNCaP-BC32 cells with or without treatment with NOS inhibitor L-NAME (100 μM). Results showed that there were more CD133^+^/CD44^+^ populations in LNCaP-BC32 cells than their parental LNCaP cells, and with such cell populations reduced upon L-NAME treatment.


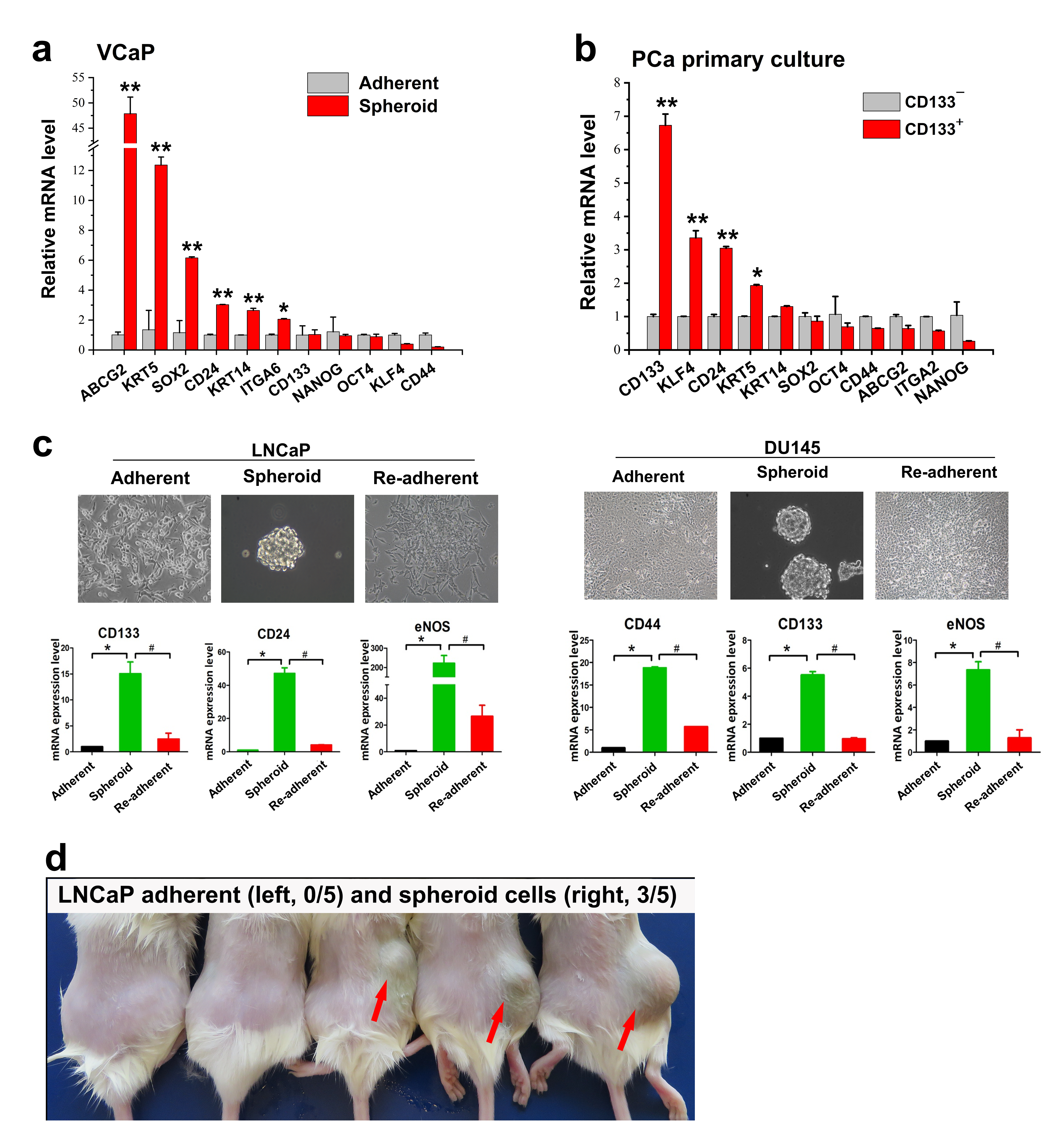


**Fig. S4.** PCSCs derived from 3D-culture spheroids and primary-cultured prostate cancer exhibit significant upregulation of eNOS. **a, b** RT-qPCR analysis of PCSC-associated markers in PCSC-enriched 3D-culture spheroids of VCaP cells and FACS-sorted CD133^+^ cells from primary-cultured prostate cancer tissues. Results showed that both the VCaP-derived 3D-culture spheroids and FACS-sorted CD133^+^ primary-cultured prostate cancer cells expressed significant higher levels of multiple PCSC/CSC-associated biomarkers. **c** RT-qPCR analysis of CSC-associated markers and eNOS in 2D-culture adherent prostate cancer cells and their corresponding 3D-culture spheroids (100x magnification). Results showed that both the LNCaP- and DU145-derived 3D-culture spheroids expressed significant higher transcript levels of CSC-associated markers (CD133, CD24 and CD44) and eNOS than their corresponding 2D-cutlture adherent cells. The expression levels of these markers and eNOS became attenuated in re-adherent 2D-culture cells derived from the 3D-culture spheroids. **d** *In vivo* tumorigenicity assay of LNCaP cells derived from adherent 2D-culture or non-adherent 3D-culture spheroids. Results showed that LNCaP cells derived from the 3D-culture spheroids (tumor formed indicated by red arrows) showed higher tumorigenicity capacity in host SCID mice than the adherent 2D-culture cells. Injected cell numbers: 10^4^ cells/injection site. Results are expressed as mean ± SD, n ≥ 3; **P* < 0.05, ***P* < 0.01.


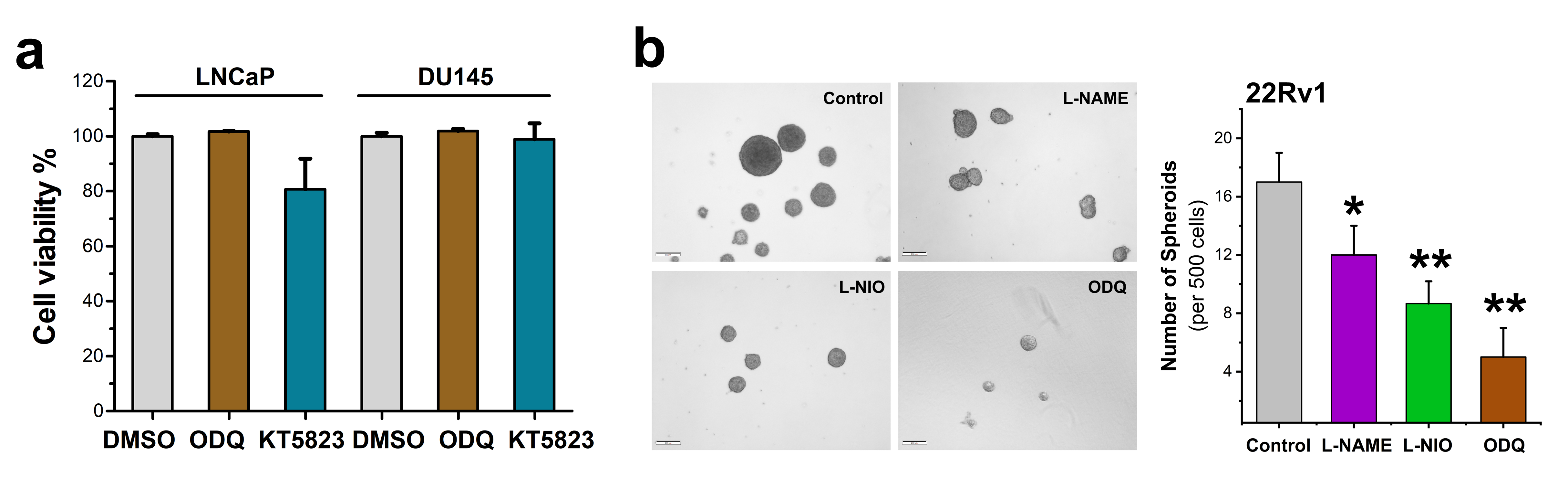


**Fig. S5.** Regulation of growth of PCSCs by eNOS is PKG-dependent. **a** Growth responses of LNCaP and DU145 cells upon toward inhibitors of sGC (ODQ) and PKG (KT5823). Results showed that treatments with ODQ and KT5823 did not affect the cell proliferation of LNCaP and DU145 cells under adherent 2D-culture condition. ODQ, 20 μM; KT5823, 10 μM. Treatment duration: 72 hr. **b** Spheroid formation efficiency of 22Rv1 cells grown under non-adherent 3D-culture condition and upon treatments with eNOS inhibitors (L-NAME and L-NIO, 100 μM respectively) and sGC inhibitor (ODQ, 20 μM). Results showed that treatments with eNOS and sGC inhibitors could significantly suppress the spheroid formation capacity of 22Rv1 cells. Results are expressed as mean ± SD, n ≥ 3. **P* < 0.05, ***P* < 0.01. Bar: 200 μm.

**Table S1**. The primers used for RT-qPCR.


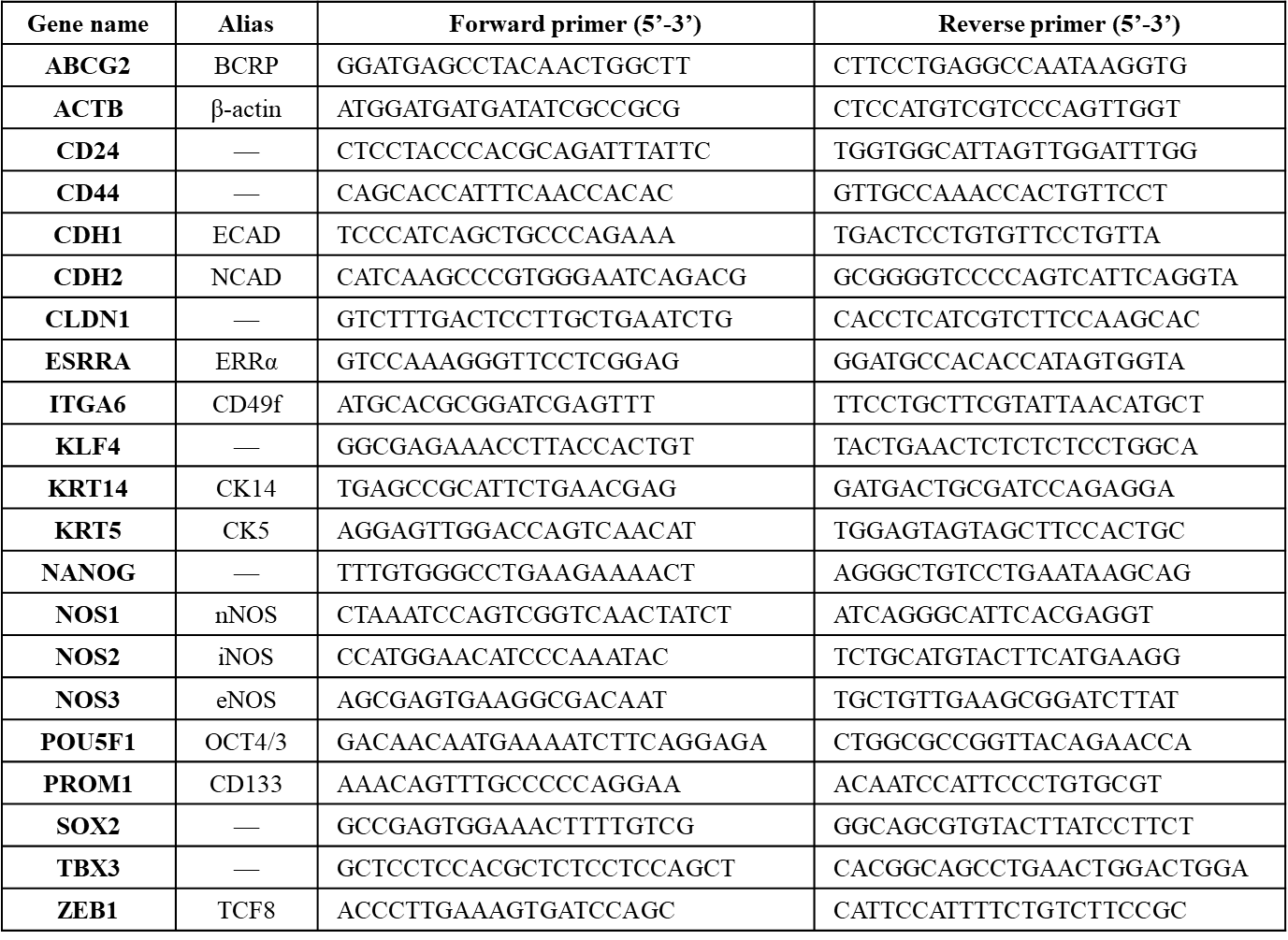


**Table S2.** The sequence of shRNAs used for eNOS knock-down.


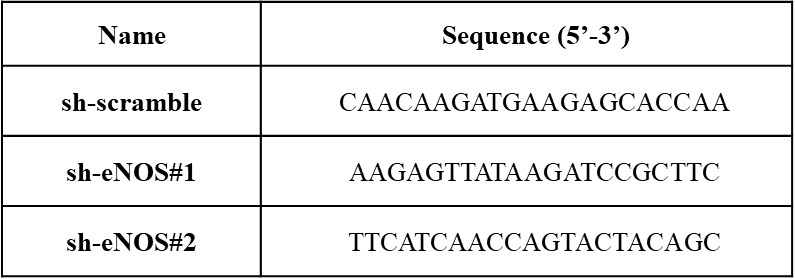

Supplement: Supplementary file 1 — Additional file 1. Supplementary Figures S1-S5 and Supplementary Tables S1 and S2. [file 13287_2022_2864_MOESM1_ESM.docx]
